# A complex association between DNA methylation and gene expression in human placenta at first and third trimesters

**DOI:** 10.1371/journal.pone.0181155

**Published:** 2017-07-13

**Authors:** Yen Ching Lim, Jie Li, Yiyun Ni, Qi Liang, Junjiao Zhang, George S. H. Yeo, Jianxin Lyu, Shengnan Jin, Chunming Ding

**Affiliations:** 1 Key Laboratory of Laboratory Medicine, Ministry of Education of China, School of Laboratory Medicine and Life Science, Wenzhou Medical University, Wenzhou, Zhejiang, China; 2 KK Women’s and Children’s Hospital, Singapore, Singapore; Beijing Cancer Hospital, CHINA

## Abstract

The human placenta is a maternal-fetal organ essential for normal fetal development and maternal health. During pregnancy, the placenta undergoes many structural and functional changes in response to fetal needs and environmental exposures. Previous studies have demonstrated widespread epigenetic and gene expression changes from early to late pregnancy. However, on the global level, how DNA methylation changes impact on gene expression in human placenta is not yet well understood. We performed DNA methylome analysis by reduced representation bisulfite sequencing (RRBS) and gene expression analysis by RNA-Seq for both first and third trimester human placenta tissues. From first to third trimester, 199 promoters (corresponding to 189 genes) and 2,297 gene bodies were differentially methylated, with a clear dominance of hypermethylation (96.8% and 93.0% for promoters and gene bodies, respectively). A total of 2,447 genes were differentially expressed, of which 77.2% were down-regulated. Gene ontology analysis using differentially expressed genes were enriched for cell cycle and immune response functions. The correlation between DNA methylation and gene expression was non-linear and complex, depending on the genomic context (promoter or gene body) and gene expression levels. A wide range of DNA methylation and gene expression changes were observed at different gestational ages. The non-linear association between DNA methylation and gene expression indicates that epigenetic regulation of placenta development is more complex than previously envisioned.

## Introduction

The human placenta is a temporary maternal-fetal organ essential for normal fetal development. It serves several functions such as exchange of oxygen, nutrients and waste products between the mother and fetus. During pregnancy, the human placenta undergoes tremendous changes in size, morphology and structure to cope with the development of the fetus [1–3].

Not surprisingly, extensive molecular changes occur during placenta development. A number of studies have investigated gene expression profiles at different structural locations of the placenta [4], and at different gestational ages of the placenta, with gene expression changes often correlating with functional changes at different gestational ages [5–8]. However, the molecular mechanisms underlying such drastic gene expression changes remain to be elucidated.

Epigenetics is considered as a fundamental mechanism regulating gene expression during development. The placenta has long been a favourite organ for the study of epigenetics, particularly in genomic imprinting [9–15]. Epigenetics is also widely considered as a mechanism for environmental factors to impact on development. For this reason, studying the epigenetics of the human placenta such as DNA methylation is particularly interesting as the placenta serves as the portal for the fetus to experience the external environment. Aberrant DNA methylation in placenta was found to be associated with pregnancy complications such as preclampsia [16,17], IUGR [18] and fetal abnormalities [19]. Recent work by others have investigated the DNA methylation changes of the placenta at different gestational ages [20], with main focus made on promoter regions.

In this study, we systematically analysed the transcriptomes and the DNA methylomes of human placenta samples derived from different gestational ages. Furthermore, we studied the dynamic correlations between gene expression and DNA methylation at different gestational ages and genomic locations.

## Materials and methods

### Ethics statement

Informed written consent was obtained under the ethics approval from the SingHealth CRIB Committee.

### Clinical samples

Women with euploidy pregnancies who attended KK Women’s and Children’s Hospital, Singapore, were recruited.

Chorionic villus samples from subjects at the first or early second trimesters of pregnancy were collected by chronic villus sampling (CVS). Placenta villi samples (fetal side) were collected from third trimester of pregnancy after delivery. All tissue samples were washed with diethylpyrocarbonate (Sigma-Aldrich, USA) treated water. For DNA analysis, tissues were stored at -80°C. For RNA analysis, tissues were incubated with RNA*later* (Life Technologies, USA) at 4°C overnight, and then stored at -80°C. Genomic DNA extraction from tissues was performed with QIAamp DNA Mini Kit (QIAGEN GmbH, Germany), according to manufacturer’s instructions. Total RNA was extracted from frozen tissues using TRIZOL protocol (Life Technologies).

### Reduced representation bisulfite sequencing (RRBS)

Six DNA samples from first trimester of pregnancies reported previously [19] and five samples from third trimester of pregnancies carrying normal fetuses were chosen for DNA methylation analysis by RRBS (S1 Table), following previously described method [19,21]. Briefly, 1–5 μg of genomic DNA was used for each library preparation. Each DNA sample was sequentially digested by MspI and Taq^α^I (New England Biolabs). The digested product was purified with the QIAquick PCR Purification Kit (QIAGEN GmbH), and was end-repaired, 3’-end-adenylated, and adapter-ligated using reagents from ChIP-Seq Sample Preparation Kit (Illumina, USA), except that the methylation adapter oligonucleotides were used in the adapter-ligation step. Two different sizes of fragments (150–197 bp and 207–230 bp) were selected by gel electrophoresis, and were then bisulfite treated using the EZ DNA Methylation-Gold Kit (Zymo Research, USA). The converted DNA was amplified using HotStarTaq DNA Polymerase Kit (QIAGEN GmbH), with 1x reaction buffer, 1.5 mM of additional MgCl_2_, 300 μM of dNTP mix, 500 nM each of PCR primer PE 1.0 and 2.0, and 2.5 U of HotStarTaq DNA polymerase. The thermocycling condition was 15 min at 94°C for heat activation, and 8–12 cycles of 20 sec at 94°C, 30 sec at 65°C and 30 sec at 72°C, followed by a 5 min final extension at 72°C. The amplified fragments were purified by gel electrophoresis and further quantified by the Agilent 2100 Bioanalyzer (Agilent Technologies, USA). Each DNA library was analyzed by two lanes of paired-end sequencing (2 × 36 bp) read on an Illumina Genome Analyzer II_x_. The paired-end 36 bp reads were analyzed using in-house developed pipeline, as previously described [19,21].

### Differential DNA methylation analysis

A total of 1,707,910 autosome CpGs with sequencing depth ≥10 and covered in at least 3 first and 3 third trimester samples were used in all subsequent analyses. Differential methylation analysis was mainly performed at regional levels. Core promoters are defined as 1kb upstream and +500bp downstream from a transcription start site while a genomic region is generated by merging nearby CpGs of less than 500bp together.

A 2-sided Mann Whitney U test was first performed at single CpG level and *p* values were adjusted within regions using Benjamini Hochberg. A promoter was considered significantly differentially methylated if 1) methylation difference between average first trimester and third trimester samples was at least 10% and 2) contained at least 2 CpGs with FDR corrected p < 0.05. For gene bodies, in additional to the above two criteria, we require that all significantly differential methylated fragments mapped to the gene body be regulated in the same direction (either all hypermethylated or hypomethylated).

All statistical analyses were performed using R package.

### mRNA sequencing (mRNA-SEQ)

Five RNA samples from first and second trimesters of pregnancies reported previously [19] and 4 samples from third trimester of pregnancies carrying normal fetuses were chosen for mRNA-seq analysis (S1 Table). Briefly, 2–5 μg of total RNA was used for each library preparation. Each RNA sample was treated with DNase I (Life Technologies). Messenger RNA purification and fragmentation, complementary DNA synthesis, end-repair, 3’-end-adenylation, and adapter-ligation were performed using Illumina’s mRNA-Seq Sample Preparation Kit. Manufacturer’s instructions were followed, except that the SuperScript III First-Strand Synthesis SuperMix (Life Technologies) was used for first strand cDNA synthesis. Adapter-ligated cDNA fragments were size-selected using a 3% agarose gel (200 ± 25 bp). The DNA samples were then amplified by PCR for 15–16 cycles. The PCR products were purified using 3% agarose gels and further quantified by the Agilent 2100 Bioanalyzer (Agilent Technologies). Each library was analyzed by one lane of either 36 bp single read or 2 × 36 bp paired-end sequencing on an Illumina Genome Analyzer II_x_.

### Differential gene expression analysis

High quality reads from RNA-seq were processed using Illumina RNA-seq pipeline, CASAVA software version 1.7, following the steps described previously [19]. The normalized gene expression level for a gene was represented by reads per kilobase per million mapped reads (RPKM) value, using the formula below:

$$RPKM=\frac{Number\;of\;aligned\;read\;for\;a\;gene\;of\;interest}{\;Number\;of\;total\;aligned\;reads\;*Transcript\;length\;for\;the\;gene\;(kb)\;}\;\times 10^{6}$$

Average RPKM values for each gene in each sample group (first and third trimester) were calculated. When the average RPKM for a gene is less than 0.5, the value was adjusted to 0.5. A gene was considered to be differentially expressed between first and third samples when: 1) two-sided Mann Whitney U test p-value less than 0.05; and 2) the ratio of (Average third trimester/Average first trimester) ≥ 2 or ≤ 0.5. R package was used for all statistical analyses.

### Effect of promoter methylation on gene expression determined by dual luciferase assays

Selected gene promoters were PCR amplified with target-specific primers (S2 Table) and cloned into pGL3-Basic Luciferase Reporter Vector (Promega) with appropriate restriction enzyme digestions. Five microgram of each promoter construct was treated with 32 units of M.*Sss*I methyltransferase (New England Biolabs) for 1.5 hours at 37^°^C and purified by the QIAquick PCR Purification Kit (QIAGEN GmbH). HEK293FT cells (Life Technologies) were cultured in Dulbecco’s Modified Eagle Medium (Life Technologies) supplemented with 10% FBS. Twenty-four hours before transfection, 1 X 10^5^ HEK293FT cells were plated into 24-well plates with 500 μl medium in each well. DNA mixture used for transfection contained 450 ng of individual promoter constructs with or without M.*Sss*I treatment or pGL3-Basic empty vector control, and 50 ng *Renilla* plasmid DNA. The DNA mixture was transfected into each well of a 24-well plate containing pre-plated HEK293FT cells in duplicates, using Jetprime transfection reagent (Polyplus-transfection SA), and media were changed 4 hours after addition of transfection reagents. Cells were harvested 48 hours post-transfection, and lysed in 100 μl passive lysis buffer (Promega) for 15 minutes at room temperature. Twenty microliter of the lysate was loaded and subjected to luciferase and renilla activity measurements on a luminometer (Promega, Glomax multi-detection system). Firefly luciferase activity was divided by *Renilla* luciferase activity to normalize for transfection efficiency. Ratio of luciferase:*Renilla* activity of experimental wells was normalized to empty vector control by subtracting value of luciferase:*Renilla* ratio of empty vector from luciferase:*Renilla* ratio of gene promoter construct. Each assay was repeated 3 times.

## Results

Using an improved method of reduced representation bisulfite sequencing (RRBS) [19,21], we quantified DNA methylation of six first trimester and five third trimester placenta villi samples. Using a minimum sequencing depth of 10 as the cutoff, we obtained on average 1.8 million CpGs per sample (S1 Table and S1 Fig). To facilitate cross gestation comparison, we further removed CpG sites that were on the sex chromosomes or present in less than three samples in either the first or the third trimester group, resulting in 1.7 million CpG sites for further analysis. These CpGs represented about 3% of Hg19 autosomal CpGs, 78% of CGIs, 70.8% of core promoters (defined as -1kb upstream and +500bp downstream from a transcription start site) and 64.2% of gene bodies (defined as +1kb downstream from a transcription start site to the transcription termination site).

**Fig 1:**
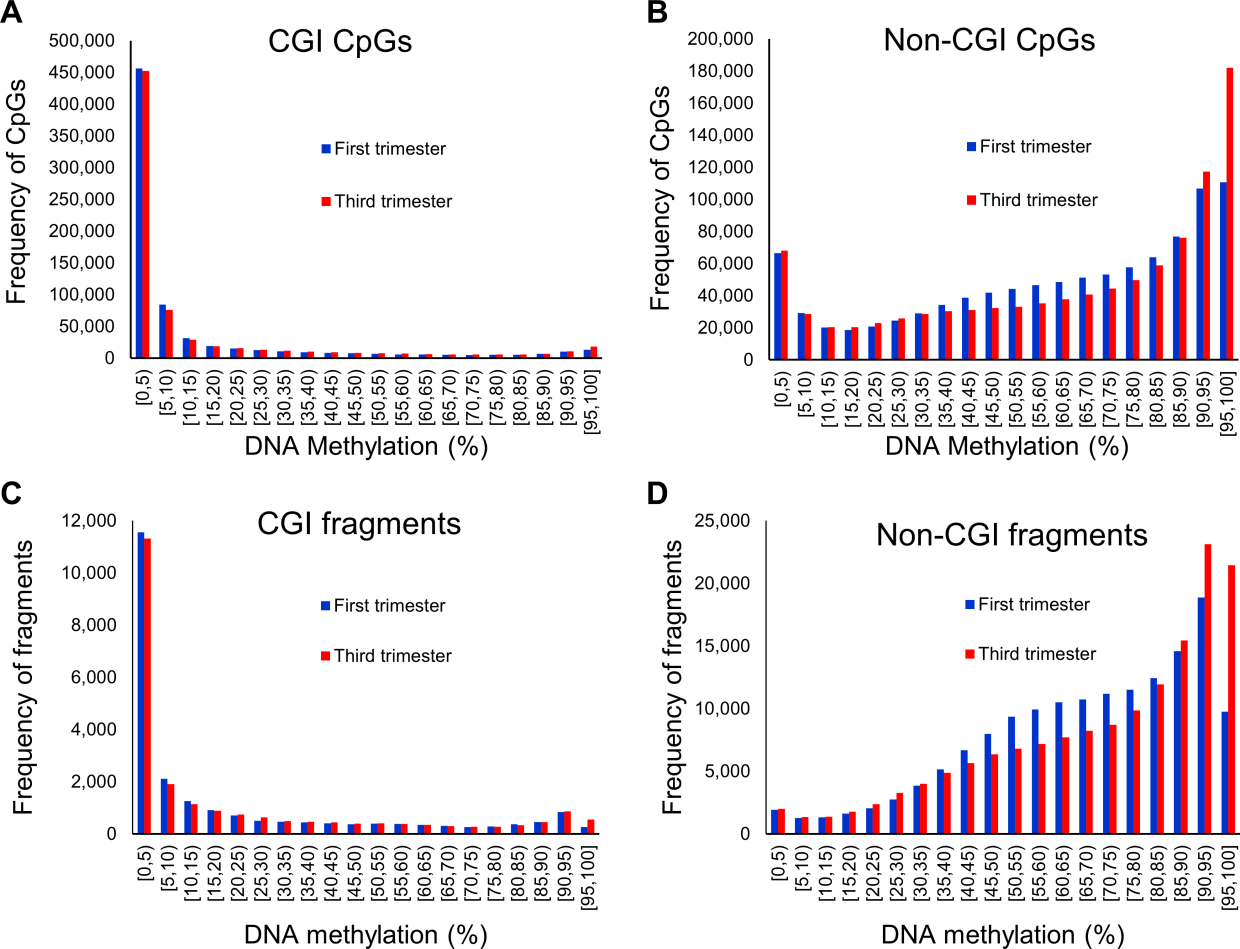
DNA methylation profiles of single and regional autosomes CpGs in CGIs and non-CGIs. Regions are created by merging nearby CpGs of less than 500bp together. (A) Distribution of the average DNA methylation by gestational age for single CpGs (723,727 CpGs sites) which lie in CGIs. (B) Distribution of the average DNA methylation by gestational age for single CpGs (984,183 CpGs sites) which do not lie in CGIs. (C) Distribution of the average DNA methylation by gestational age for regions (22,652 regions sites) which lie in CGIs. (D) Distribution of the average DNA methylation by gestational age for regions (153,563 regions) which do not lie in CGIs.

The distribution of individual CpG methylation levels were drastically different for CpG island (CGI) and non-CpG island (non-CGI) regions, as were shown in many earlier studies in placenta and other cell types [19,22–26] (Fig 1A and 1B). Similar distribution was observed for DNA fragments from merging neighbouring CpGs (Fig 1C and 1D).

**Fig 2:**
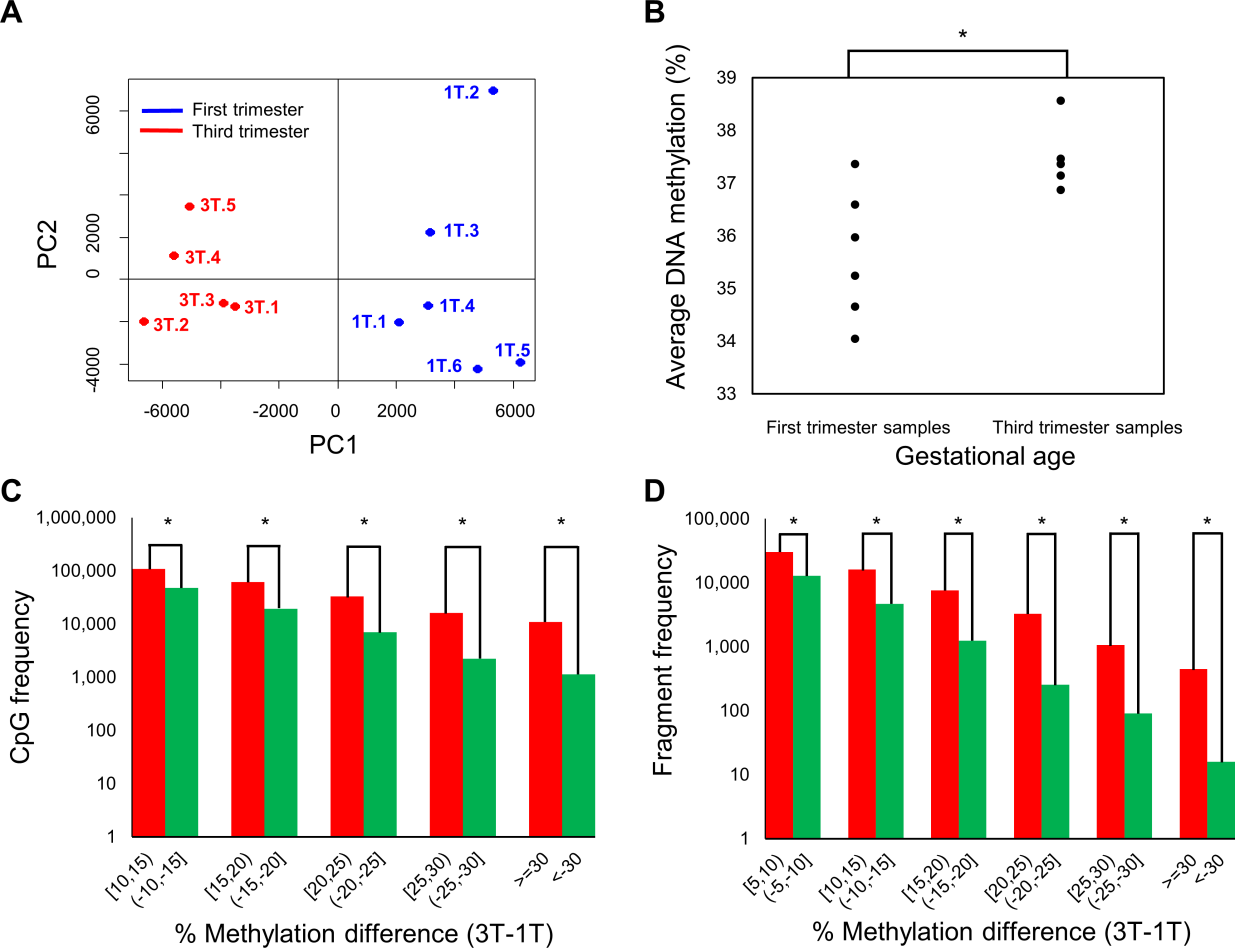
DNA methylation distinguishes samples by gestational age. (A) PCA plot on 1.7 million autosome CpGs shows separation between first and third trimester samples. (B) Dot plot of the average DNA methylation for each sample using 730,594 common CpGs across 11 samples. The third trimester samples show higher DNA methylation than the first trimester samples. Mann-Whitney U test on the average DNA methylation values show significant differences (p = 0.028). (C) Distribution of the difference in average DNA methylation between first and third trimester samples, at single CpG level. Strong evidence of hypermethylation was observed, supported by higher peaks in the red bars for all bins of DNA methylation difference. (D) Distribution of the difference in average DNA methylation between first and third trimester samples, at regional level. A similar hypermethylation observation in Fig 2C was observed at regional level.

In non-CGI regions, there was an apparent enrichment of highly methylated CpGs (95–100% methylation) in the third trimester samples (Fig 1B and 1D), indicative of global differences in DNA methylation at different gestation ages. Principal component analysis showed distinct separation of samples based on gestation age (Fig 2A). Additionally, there was a significant increase in mean CpG methylation in third trimester (*p* = 0.028, 2-sided Mann-Whitney U test) (Fig 2B). Furthermore, at both individual CpG and genomic fragment level, hypermethylation was consistently more frequent than hypomethylation (Fig 2C and 2D).

DNA methylation changes in promoters and gene bodies were further analysed as methylation of these regions have been demonstrated to be associated with gene expression. A total of 199 promoters (corresponding to 189 genes) were found to be significantly differentially methylated between the first and third trimester samples, with 193 (183 genes) (96.8%) showing higher methylation in the third trimester group. We also identified 2,297 gene bodies to be significantly differentially methylated, with 2,136 (93.0%) being hypermethylated in the third trimester samples.

**Fig 3:**
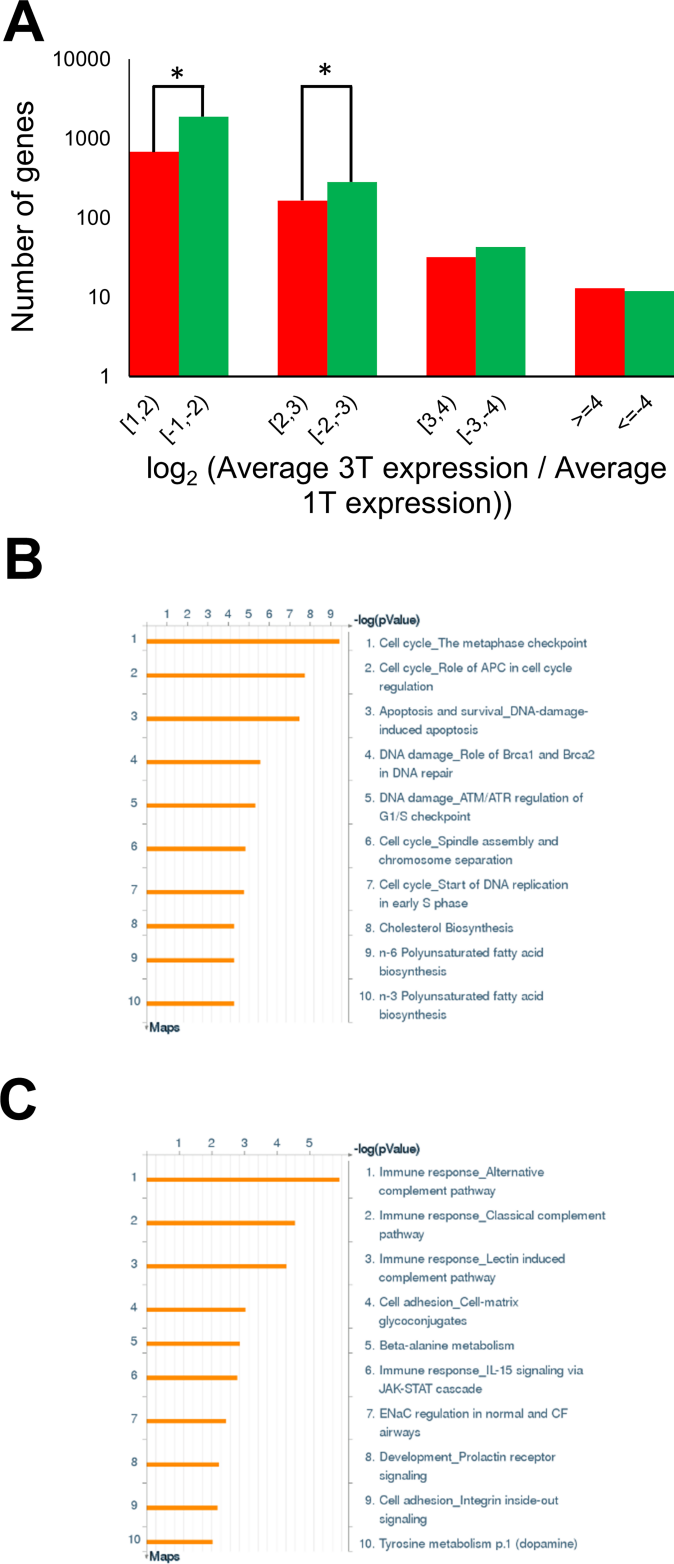
Expression changes during placental development across gestational age. (A) Distribution of gene expression changes from first to third trimester for the genes that showed at least 2 fold changes. There is a general gene repression from first to third trimester, indicated by higher green bars. (B) Metacore analysis on the repressed genes at third trimester. Genes were mostly involved in cell cycle. (C) Metacore analysis on the activated genes at third trimester. Genes were mostly involved in immune response and signalling pathways.

We next carried out RNA-Seq analysis on five first/second trimester and four third trimester placenta villi samples (S1 Table). Genes with low expression levels (RPKM < 0.5) in both sample groups were filtered out, leaving 13,756 genes for differential gene expression analysis. A total of 2,447 genes were significantly differentially expressed between first/second and third trimester samples, of which, 1,889 (77.2%) were down-regulated and 588 (22.8%) were up-regulated in the third trimester samples (Fig 3A). Gene ontology analysis with multiple testing correction (p < 0.05) using a commercial database (MetaCore from GeneGo Inc.) was performed separately on the down-regulated (Fig 3B) and up-regulated gene lists (Fig 3C). The down-regulated genes were enriched mainly in the cell cycle pathways, with the top three pathways being “Cell cycle_The metaphase checkpoint”, “Cell cycle_Role of APC in cell cycle regulation” and “Apoptosis and survival_DNA-damage-induced apoptosis”. The up-regulated genes, on the other hand, were mainly related to immune response, with the top three pathways being “Immune response_Alternative complement pathway”, “Immune response_Classical complement pathway” and “Immune response_Lectin induced complement pathway”.

Eleven imprinted genes were also differentially expressed, of which ten (*SLC22A18*, *PEG10*, *MEST*, *NAP1L5*, *MIMT1*, *PSIMCT-1*, *PEG3*, *LIN28B*, *DGCR6*, *PLAGL1*) showed higher expression level for the first trimester samples and one (*ANO1*) showed higher expression in the third trimester (Table 1).

**Table 1:** List of the imprinted genes showing expression changes between first and third trimester. The list was obtained from http://www.geneimprint.com. Expression was given relative to the third trimester samples.

Gene	Average first trimester (RPKM)	Average third trimester (RPKM)	log_2_ (3^rd^ trimester/1^st^ trimester)	Maternal/Paternal imprinted
SLC22A18	23.25	3.90	-2.58	Maternal
PEG10	612.10	188.34	-1.70	Paternal
MEST	291.60	93.04	-1.65	Paternal
NAPIL5	4.26	1.48	-1.53	Paternal
MIMT1	1.34	0.50	-1.42	Paternal
PSIMCT-1	3.246	1.35	-1.26	Paternal
PEG3	194.33	81.51	-1.25	Paternal
LIN28B	23.84	10.08	-1.24	Paternal
DGCR6	1.38	0.64	-1.11	Unknown
PLAGL1	119.87	56.75	-1.08	Paternal
ANO1	3.90	8.07	1.05	Maternal

**Fig 4:**
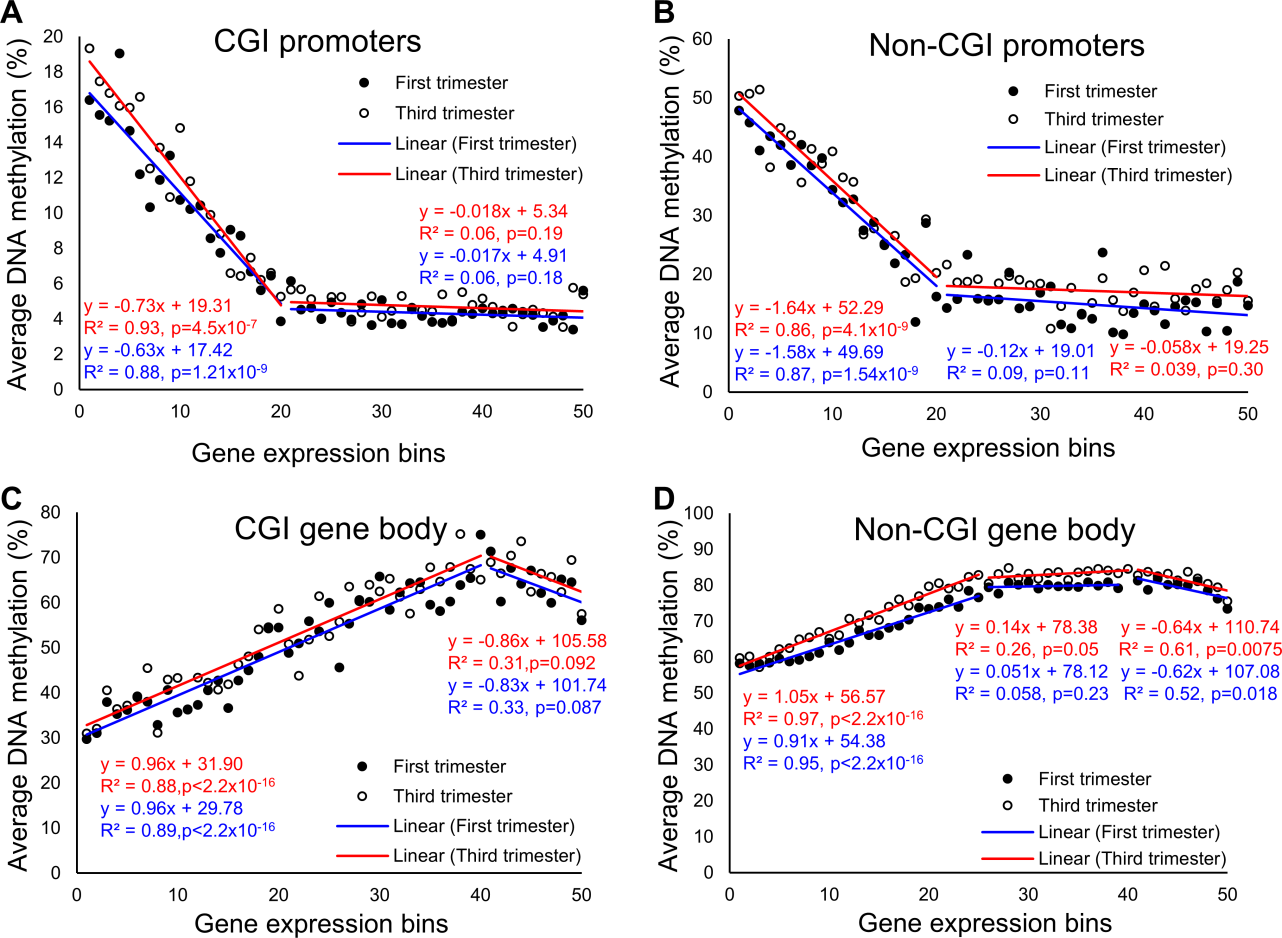
Correlation between DNA methylation and gene expression. **Genes were grouped into 50 bins, in order of increasing gene expression. DNA methylation of the promoter or gene body fragments within each gene expression groups were then averaged to obtain the relationship.** (A) Scatterplot of the DNA methylation of promoters in CGI against the gene expression showed anti-correlation for the lower expressed genes. (B) Scatterplot of the DNA methylation of promoters in non-CGI against the gene expression showed anti-correlation for the lower expressed genes. The non-CGI promoters showed higher DNA methylation than the CGI promoters. (C) Scatterplot of the DNA methylation of gene body fragments in CGI against the gene expression shows positive correlation. (D) Scatterplot of the DNA methylation of gene bodies in non-CGI against the gene expression shows positive correlation. The non-CGI gene bodies showed higher DNA methylation than the CGI gene bodies.

To explore the correlation between DNA methylation and gene expression, we first separated the genes into 50 equal size bins with increasing expression levels. The average DNA methylation level of the promoters in each bin was then calculated. Interestingly, for both CGI promoters (promoters overlapping with CGIs) and non-CGI promoters, a non-linear correlation between DNA methylation and gene expression was observed (Fig 4A and 4B). There was a clear anti-correlation for genes with lower expression levels (expression bins 1 to 20). However, for genes at higher expression levels (bins 21 and above), DNA methylation levels were largely similar regardless of gene expression level.

The correlation between DNA methylation at gene bodies and gene expression was also non-linear, with CGI gene bodies and non-CGI gene bodies behaving somewhat differently (Fig 4C and 4D). For CGI gene bodies, there was a positive correlation between DNA methylation and gene expression, for genes in bin 1 to bin 40, followed by a seemingly negative correlation for genes in bin 41 to 50. For non-CGI gene bodies, the positive correlation was only observed for genes in bin 1 to bin 25. For genes in bin 26 to about 40, DNA methylation levels were largely similar. Similarly, there was a seemingly negative correlation for genes in bin 41 to 50 (Fig 4A–4D). We did not observe a difference in exonic and intronic regions (S2 Fig). However, the DNA methylation levels of CGI exons were consistently higher than CGI introns, regardless of gene expression levels (S3A and S3B Fig).

**Fig 5:**
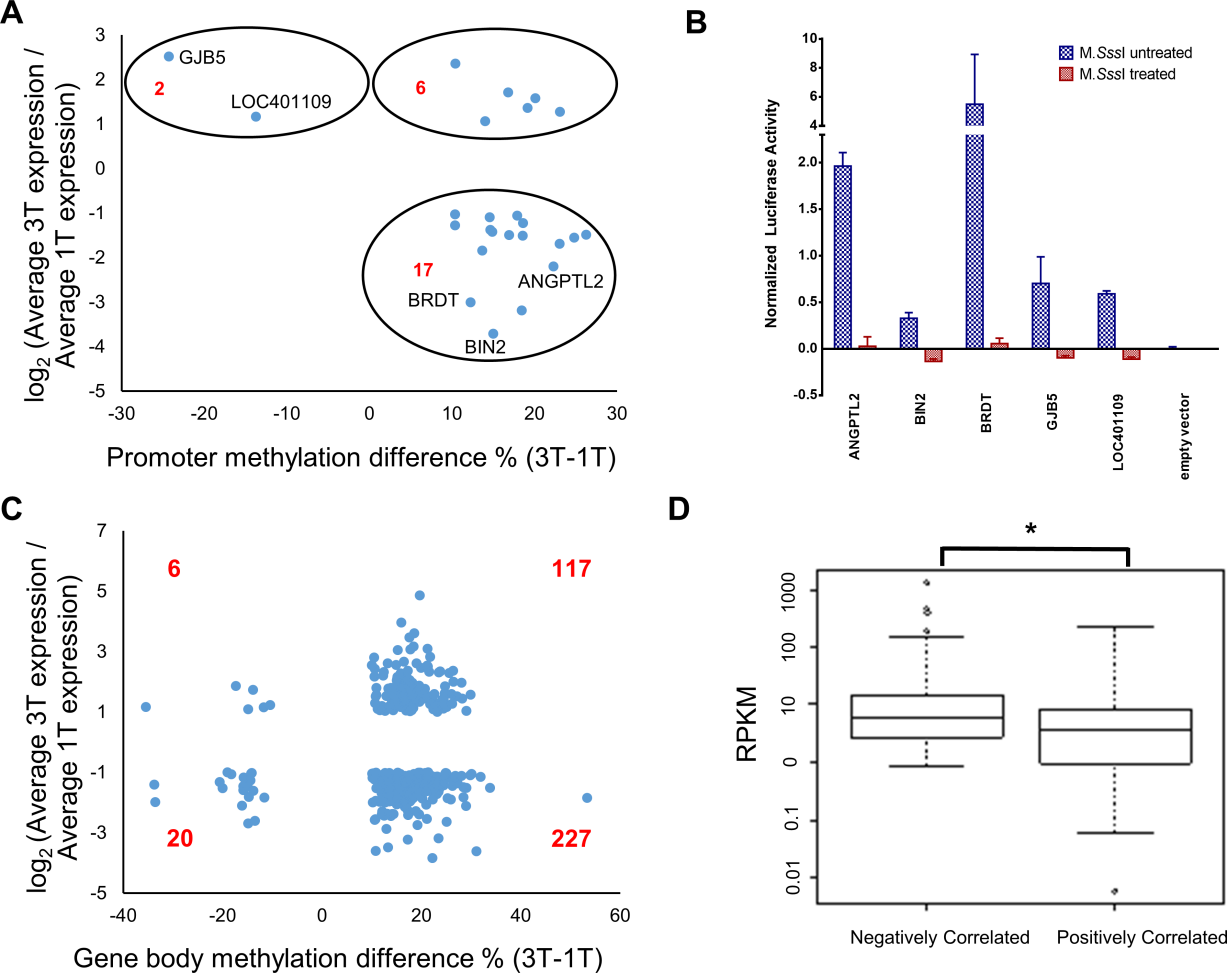
Correlation between significant differential DNA methylation and differential gene expression. (A) Scatterplot of differentially methylated promoters with differential gene expression between first and third trimester samples. 19 out of 25 promoters showed anti-correlation. (B) Experimental validation of the genes labelled in Fig 5A, showing DNA methylation is associated with gene repression. Dual luciferase assays were performed, using empty vector as negative control. (C) Scatterplot of differentially methylated gene bodies with differential gene expression between first and third trimester samples. Majority of the genes showed negative correlation. (D) The differential expressed and methylated gene bodies from Fig 3C were grouped by positive and negative correlation. A boxplot comparing the initial gene expression at first trimester was given. The negatively correlated group showed elevated gene expression compared to the positively correlated group (2 sided p-value = 8.38*10^−6^).

Lastly, we asked how changes in DNA methylation from early gestation to late gestation in human placenta affects gene expression. A total of 25 genes (***Table 2***) showed both differential gene expression and differential DNA methylation in promoters when comparing the two gestation groups (Fig 5A). Of those, 19 genes (78%) showed anti-correlation between changes in gene expression and changes in promoter DNA methylation. There was a statistically significant difference between the positive and negative correlations (2-sided binomial test, *p* = 0.015). We validated the results in five genes (*GJB5*, *LOC401109*, *BRDT*, *BIN2* and *ANGPTL2*) using the dual luciferase assay by cloning the respective promoters into the reporter vectors. Gene expression repression was observed in all five genes when the vectors were treated with the methyltransferase M.SssI (Fig 5B).

**Table 2:** List of genes having both significant promoter DNA methylation difference and significant gene expression between first and third trimester placenta samples.

Gene	Promoter DNA methylation change (3T-1T)	Gene Expression (log_2_(3T/1T))
ANGPTL2	22.32	-2.20
BIN2	18.44	-3.19
BRDT	12.26	-3.01
CCRL2	23.06	-1.69
CYP2W1	15.00	-3.72
FAM111A	14.58	-1.10
FBXO17	16.93	-1.49
FGL2	26.25	-1.49
GJB5	-24.29	2.51
GREB1	17.90	-1.06
HSPB2	10.37	-1.28
ISLR	18.62	-1.23
LOC100289019	14.03	1.06
LOC401109	-13.71	1.16
MAL	10.43	2.36
PLEKHA6	20.11	1.58
PTPRE	10.36	-1.03
RAB42	18.59	-1.51
RHOBTB2	14.66	-1.38
SEMA6D	24.79	-1.56
SNORD110	13.68	-1.85
SNRPF	14.89	-1.43
ST5	19.15	1.36
STRA6	16.80	1.71
SYNPO	23.10	1.27

A total of 370 genes showed both differential gene expression and differential DNA methylation in gene bodies when comparing the two gestation groups (Fig 5C). Of those, 233 (63%) showed negative correlation between changes in gene expression and changes in gene body DNA methylation. Similar to the promoters, there was a statistically significant difference between the positive and negative correlations (2-sided binomial test, *p* = 6.85 x 10^−7^). Given that the correlation between gene body methylation and gene expression was positive for genes with relatively lower expression, but negative for genes with higher expression (Fig 4C and 4D), we hypothesized that the negatively correlated genes (233 genes) were of higher expression levels than the positively correlated genes. RNA-seq data confirmed our hypothesis (Fig 5D).

## Discussion

In this study, we applied next generation sequencing techniques to study the gene expression (by RNA-Seq) and DNA methylation profiles (by RRBS) of human placenta tissues derived from early and late gestations.

A total of 2,477 genes, including 11 imprinted genes, were found to be differentially expressed between the early and late gestational age placenta samples (Table 1).

Imprinted genes are essential to the normal growth and development of the mammalian fetus. Paternally and maternally expressed genes have been known to promote and repress fetal growth, respectively.[27]. Alterations in imprinted genes have been implicated in pregnancy complications such as intrauterine growth restriction (IUGR) [28,29], preeclampisa (PE) [30,31] and lethality [32,33]. Prospectively, even if the fetus survives to birth, these effects may be exhibited chronically and are linked to increased risks for hypertension [34], cardiovascular disease [35–37], abnormalities in neuro [38] and renal development [39,40].

Our RNA-seq results shows that eleven imprinted genes were significantly differentially expressed, of which eight were paternally expressed, two were maternally expressed and the remaining one being random. All of the eight paternally expressed genes were down-regulated in the third/term trimester, which is coherent with the fully developed status of the fetus.

Gene ontology analysis revealed that down- and up-regulated genes during placenta development were associated with cell cycles and immune responses, respectively.

An overall DNA hypermethylation was observed in placental tissues at later gestational ages, consistent with earlier work using a much lower throughput method (Illumina Infinium HumanMethylation 27 Beadchip) [20]. The overall DNA hypermethylation changes were observed in both promoters and gene bodies, which also coincided with largely gene expression repression.

The association between DNA methylation and gene expression was found to be complex and dependent on at least two factors: genomic context (promoters or gene bodies) and gene expression level. Consistent with previous findings from placenta [20] and other biological systems [41–43], we found a negative correlation between gene expression level and promoter methylation level in both early and late pregnancies, as well as a positive correlation between gene expression level and gene body methylation level [24,44–46]. However, these correlations were no longer present in genes with higher expression levels (Fig 5). In contrast, for genes with the highest expression levels, there was a negative correlation between gene expression and DNA methylation (Fig 5).

There are a few limitations in our study. First, the placenta is a complex organs with different cell types at different structural locations with different expression profiles [4]. Secondly, placenta samples from additional time points during pregnancy may provide more dynamic and detailed changes in gene expression and DNA methylation. Thirdly, although majority of the genes with both significant differential methylation and gene expression were anti-correlated, the minority set which showed positive correlation might be regulated by other epigenetic mechanisms such as histone modification, transcriptional factor binding and nucleosome positioning.

## Supporting information

S1 Fig The histogram gives the average number of CpGs sites for 11 samples, with varying minimum sequencing depths. Error bars represents standard deviation for 11 samples.
(TIF)

S2 Fig Correlation between DNA methylation and gene expression. Genes were grouped into 50 bins, in order of increasing gene expression. DNA methylation of the exons/introns fragments within each gene expression group was then averaged to obtain the relationship. Scatterplot of the DNA methylation of gene body exons and introns against the gene expression showed positive correlation. The DNA methylation in exons and introns did not exhibit clear differences.
(TIF)

S3 Fig Correlation between DNA methylation and gene expression. Genes were grouped into 50 bins, in order of increasing gene expression. DNA methylation of the exons/introns fragments within each gene expression group was then averaged to obtain the relationship. The genes were divided into 4 groups where non-CGI introns and exons showed similar pattern while differences were observed between exons and introns in CGI gene bodies. First and third trimester samples showed similar patterns and trends.
(TIF)

S1 Table Sample information. (XLSX)

S2 Table Information on dual luciferase assays on selected genes. (XLSX)
