# Supplementary figures and images for: A complex association between DNA methylation and gene expression in human placenta at first and third trimesters
Source: PLoS One. 2017 Jul 13;12(7):e0181155. doi: 10.1371/journal.pone.0181155 (PMC5509291; doi:10.1371/journal.pone.0181155)

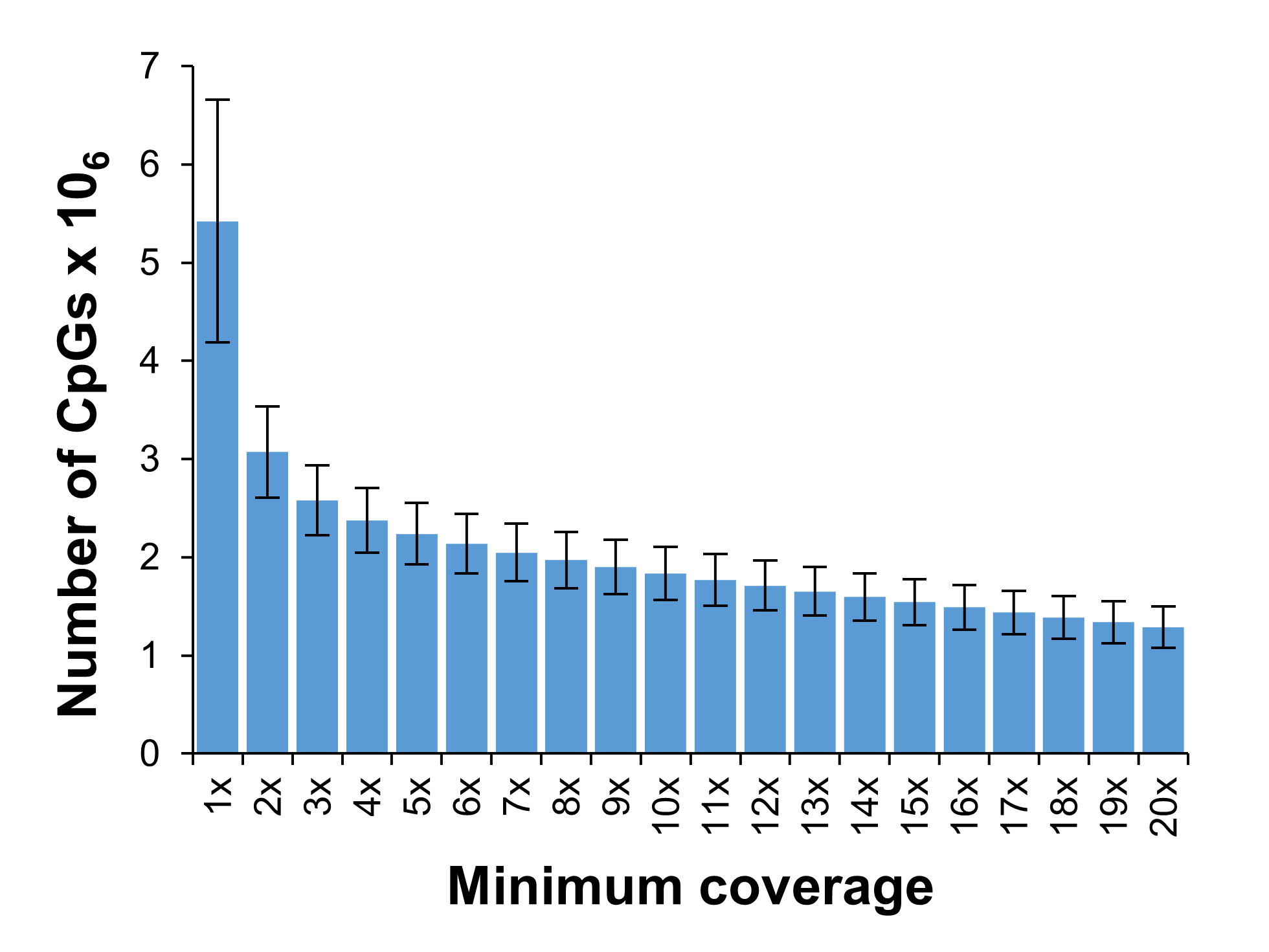

Supplement: S1 Fig — Error bars represents standard deviation for 11 samples. (TIF) [file pone.0181155.s001.tif]

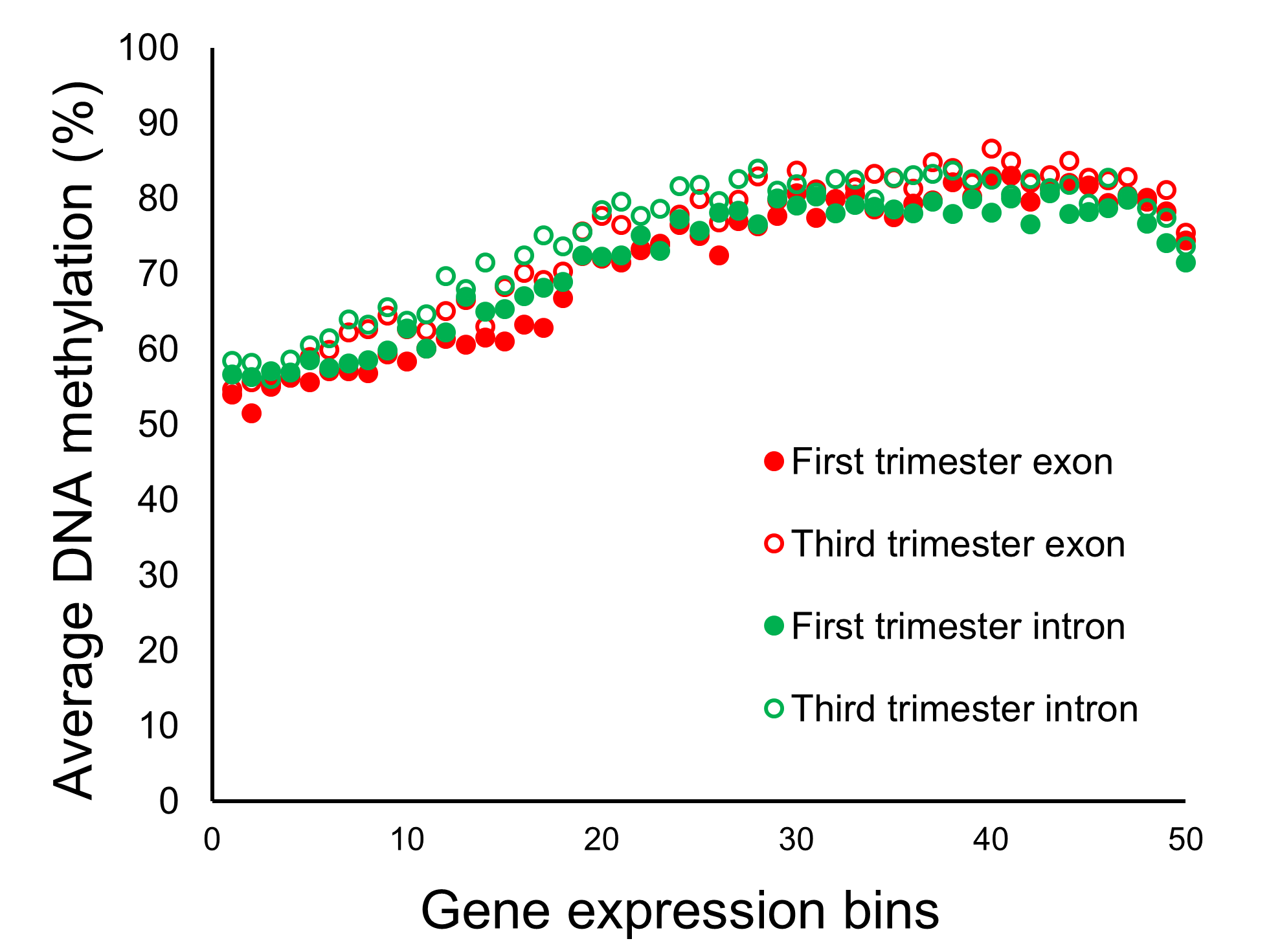

Supplement: S2 Fig — Genes were grouped into 50 bins, in order of increasing gene expression. DNA methylation of the exons/introns fragments within each gene expression group was then averaged to obtain the relationship. Scatterplot of the DNA methylation of gene body exons and introns against the gene expression showed positive correlation. The DNA methylation in exons and introns did not exhibit clear differences. (TIF) [file pone.0181155.s002.tif]

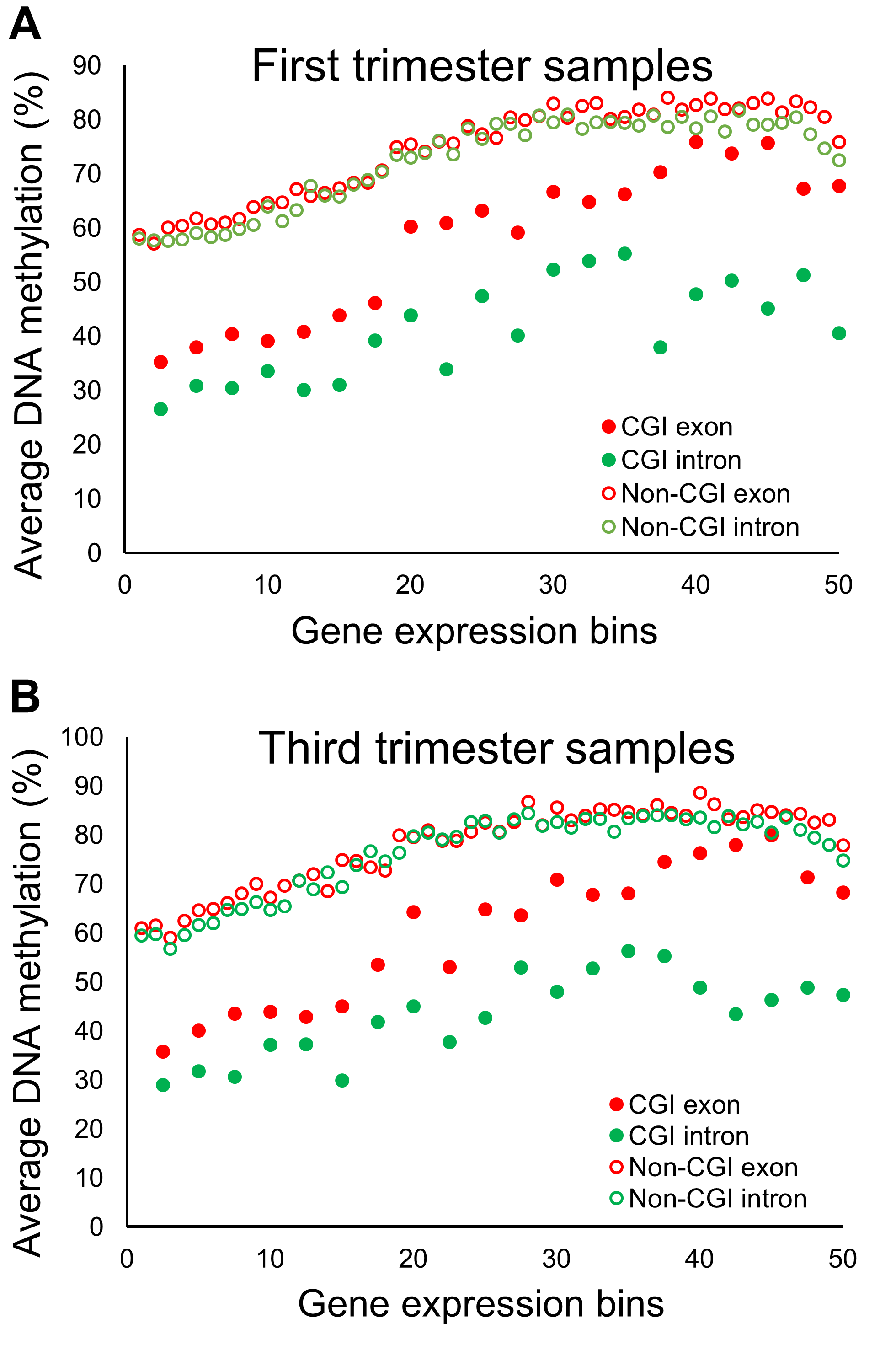

Supplement: S3 Fig — Genes were grouped into 50 bins, in order of increasing gene expression. DNA methylation of the exons/introns fragments within each gene expression group was then averaged to obtain the relationship. The genes were divided into 4 groups where non-CGI introns and exons showed similar pattern while differences were observed between exons and introns in CGI gene bodies. First and third trimester samples showed similar patterns and trends. (TIF) [file pone.0181155.s003.tif]
